# Identification of PANoptosis-relevant subgroups and predicting signature to evaluate the prognosis and immune landscape of patients with biliary tract cancer

**DOI:** 10.1007/s12072-024-10718-x

**Published:** 2024-08-10

**Authors:** Dongming Liu, Wenshuai Chen, Zhiqiang Han, Yu Wang, Wei Liu, Aomei Ling, Qiang Wu, Huikai Li, Hua Guo

**Affiliations:** 1 Department of Tumor Cell Biology, Tianjin Medical University Cancer Institute and Hospital, Tianjin, 300060 China; 2 Department of Hepatobiliary Cancer, Liver Cancer Research Center, Tianjin Medical University Cancer Institute and Hospital, Tianjin, 300060 China; 3 Department of Anesthesiology, Tianjin Medical University Cancer Institute and Hospital, Tianjin, 300060 China; 4 National Clinical Research Center for Cancer, State Key Laboratory of Druggability Evaluation and Systematic Translational Medicine, Tianjin Key Laboratory of Digestive Cancer, Tianjin’s Clinical Research Center for Cancer, Tianjin, 300060 China

**Keywords:** Apoptosis, Pyroptosis, Necroptosis, Immunotherapy, Chemotherapy, Biomarkers, Risk score, Molecular subtyping, Prognostic model, Clinical strategy

## Abstract

**Background:**

This study conducted molecular subtyping of biliary tract cancer patients based on 19 PANoptosis-related gene signatures.

**Methods:**

Through consensus clustering, patients were categorized into two subtypes, A and B. By integrating multi-omics data and clinical information from different cohorts, we elucidated the association between different subtypes of biliary tract cancer and patient prognosis, which correlated with the immune infiltration characteristics of patients.

**Results:**

LASSO regression analysis was performed on the 19 gene signatures, and we constructed and validated a 9-gene risk score prognostic model that accurately predicts the overall survival rate of different biliary tract cancer patients. Additionally, we developed a predictive nomogram demonstrating the clinical utility and robustness of our model. Further analysis of the risk score-based immune landscape highlighted potential associations with immune cell infiltration, chemotherapy, and immune therapy response.

**Conclusion:**

Our study provides valuable insights into personalized treatment strategies for biliary tract cancer, which are crucial for improving patient prognosis and guiding treatment decisions in clinical practice.

**Supplementary Information:**

The online version contains supplementary material available at 10.1007/s12072-024-10718-x.

## Background

Biliary tract cancer (BTC) is the second most common primary hepatic malignancy after hepatocellular carcinoma (HCC), and its incidence has been increasing globally over the past decades. Intrahepatic cholangiocarcinoma accounts for 20% of all BTCs, and the rest are extrahepatic cholangiocarcinomas (including perihepatic and distal cholangiocarcinoma) and gallbladder cancer [1, 2]. A typical feature of BTC is the embedding of cancer cells in a dense stroma containing fibroblasts, lymphatic vessels, and various immune cells. The functional role of the reactive tumor stroma has not been fully elucidated; however, recent research suggested that the tumor microenvironment plays a key role in the progression and aggressiveness of BTC [1, 3].

Given that the primary goal of cancer therapy is to destroy tumor cells, inducing cancer cell death is an important strategy in cancer treatment [4]. Programmed cell death (PCD) is an active mechanism for maintaining the development and survival of the organism [5, 6]. The most studied pathways of PCD are apoptosis, pyroptosis, and necroptosis [7–9], which have a crucial role in the tumor microenvironment. Recent studies identified a cell death process called PANoptosis, which include apoptosis, pyroptosis and necroptosis [10, 11]. Current studies on PANoptosis in tumors mainly focused on solid malignancies such as hepatocellular carcinoma [12–14], pancreatic carcinoma [15], lung carcinoma [16–18], and renal carcinoma [19, 20]. However, the role of PANoptosis in BTC pathogenesis has not been investigated.

This study investigated the prognostic value of PANoptosis-related genes (PRGs) and established a signature to predict prognosis and immune/chemotherapy response in patients with BTC. The clinical applicability of the signature was thoroughly evaluated and validated using external datasets. In addition, this study demonstrated that the PRGs were significantly correlated with the tumor immune microenvironment. The findings provide new insights into the development of personalized treatment regimens for patients with BTC and to improve their clinical outcomes.

## Materials and methods

### Data collection

The RNA-seq data and corresponding clinical information from GSE89749 as the training data were obtained from the Gene Expression Omnibus (GEO) database while the data downloaded from the Cancer Genome Atlas Program (TCGA) was used for external validation. 115 BTC samples were enrolled in GSE89749, 33 in TCGA.

### PRG consensus clustering analysis

Previous PANoptosis-related research has proposed and applied the 19 PANoptosis-related genes (PRGs) for further investigation, PRGs were identified from prior studies [21–29]. We conducted unsupervised consensus clustering analysis based on the expression level of 19 Panotopsis genes for patients in GSE89749 and visualized the relevant analysis results using the R package ‘ConsensusClusterPlus’, where the samples were separated into 3 primary clusters at the *k* = 3. Initially, we intended to discriminate the BTC patients according to the integral PANoptosis-related transcriptomic features so we conducted unsupervised clustering for all samples at first where the algorithm calculated on account of the overall expression of 19-PRGs rather than a single one. In other words, we took all the PANoptosis-genes in account without the elimination of several genes in PRGs with no significance among BTC patients, which can help us to discern patients by condition of PANoptosis, avoiding artificial bias. The A (cluster 1 and 2) and B (cluster 3) clusters are based on the unsupervised clustering of 19 gene expression profiles, and on the basis of this classification, we further extracted and refined the differential genes of the two clusters and then constructed the risk scores, so they are not directly correlated in the traditional sense.

### Functional enrichment analysis of differentially expressed genes (DEGs)

Kyoto Encyclopedia of Genes and Genomes (KEGG) and Gene Ontology (GO) analyses were performed to predict the metabolic pathways and gene function, respectively, using ggplot2, Bioconductor, Hs.eg.db, and org R packages. The threshold was set at *p*-value < 0.05.

### Establishment of prognostic features based on PANoptosis

Differential expressed genes in two PRG clusters were used to build the prognostic signature. The least absolute shrinkage and selection operator (LASSO) penalized Cox regression analysis was performed to establish a predicting model using the R package “glmnet”. The expressions of 9 candidate risk-associated genes were respectively weighted by their regression coefficients, calculating the individuals risk score by the following formula: ${\sum }_{i=1}^{n}\beta i* \lambda i$, where, n represents the number of genes, $\lambda i$ is the gene expression level, and $\beta i$ is the regression coefficient. The value from the maximally standardized long-rank statistics were chosen as a cutoff point to group the patients into two risk stratifications: high-risk and low-risk group. The differential overall survival for high- and low-risk patients was evaluated by Kaplan–Meier (KM) analysis using the R packages “survival” and “survimer”. Moreover, the predictive performance of the prognostic prediction model was assessed by performing the area under the curve (AUC) of the receiver operating characteristic (ROC) curve. Apart from the risk score, we also enrolled other three clinical predictors including sex, age, and whether with the primary sclerosing cholangitis (PSC) to build the predictive nomogram which can forecast the 1 year, 3 year, and 5 year overall survival for the BTC patients using the *R* package “rms”. We also plotted the calibration curves to compare the predicted against the actual survival probabilities. One randomly selected patient from GSE89749 validated the probability of 1–5 year OS using the aforementioned factors. We conducted the R package “nomogramEx” to calculate the total point. Ultimately, the interactive nomogram was depicted and visually displayed by the R package “regplot”.

### Assessment of the tumor microenvironment in high- and low-risk subgroups

The abundance of diverse immune cells infiltration was inferred and quantified using ImmuneCellAI for individuals in high- and low-risk groups, to investigate the correlation of the risk model with tumor microenvironment (TME). Additionally, we further investigated diverse immune cells infiltration abundances in different groups which were divided based on the expression level of the 9 risk genes respectively.

### Assessment of mutations, effects on immune therapy, and chemotherapeutics in high- and low-risk subgroups

We applied the R package “pRRophetic” to predict the chemotherapeutic response in high- and low-risk groups. We inferred the clinical chemotherapeutic response of each sample to 12 common agents, and the *t*-test was used to perform statistical analyses of differences.

### Statistical analysis

R version 4.1.0 software was used for statistical analysis, and |log fold change (FC)|> 2 and *p*-value < 0.05 were considered statistically significant.

## Results

### Hereditary variability of PRGs in BTC

**Fig. 1:**
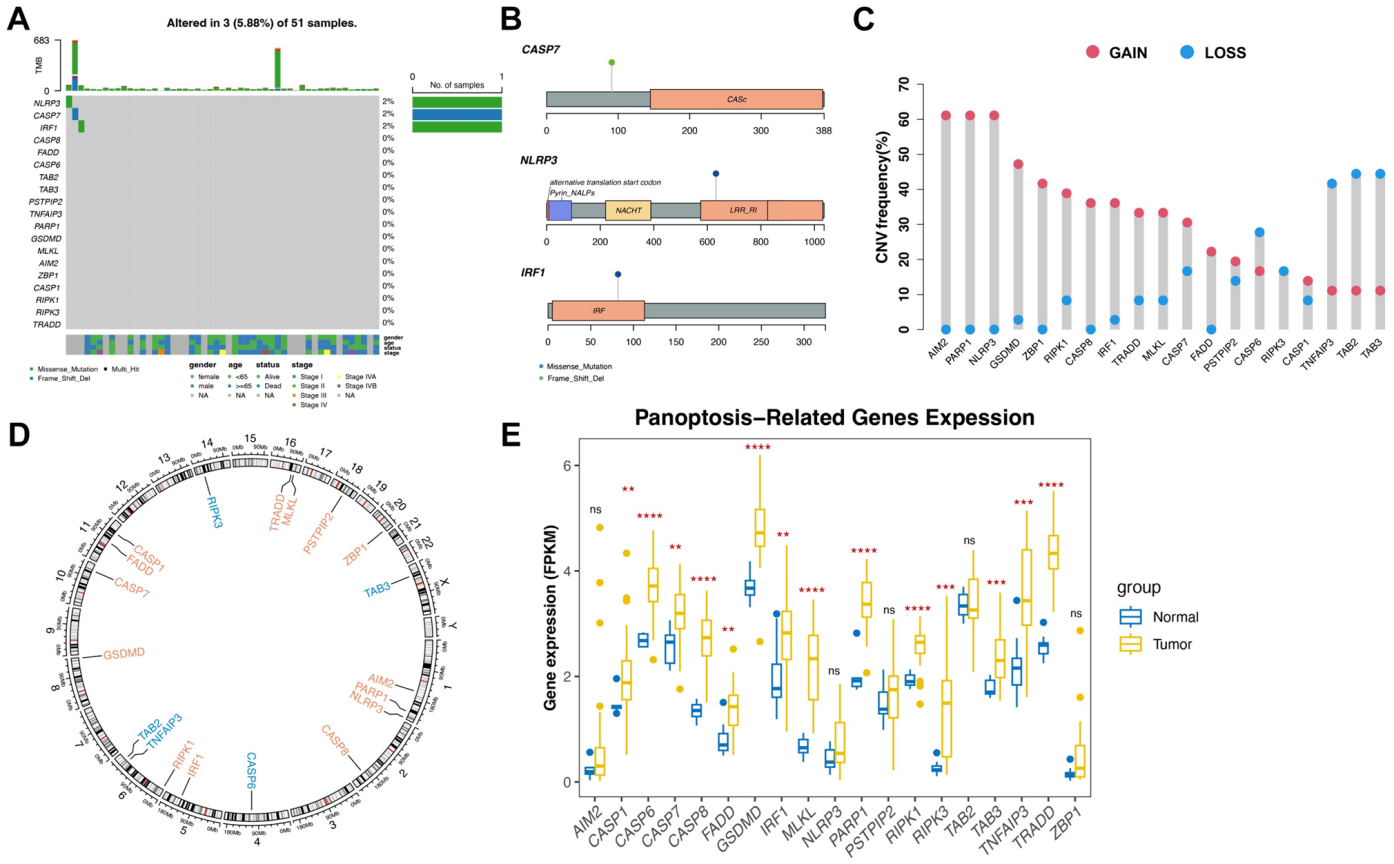
Hereditary variability of PRGs in BTC. **A** Mutation situations of 19 PRGs in patients with BTC. **B** Detailed mutation type of NLRP3, CASP7, and IRF1. **C** Copy number alterations of PRGs. **D** Locations of CNV alterations for PRGs on 23 chromosomes. **E** Expression difference of PRGs between BTC and adjacent normal samples. *p < 0.05; **p < 0.01; ***p < 0.001

A total of 33 patients with BTC were examined to reveal the hereditary variability and differential expression of PRG. A comparison of 32 BTC and 8 adjacent normal samples was also conducted. Investigation of the somatic mutagenesis of 19 PRGs in patients with BTC revealed a mutation in three PRGs (5.88%) from 51 samples, and the highest mutation frequency was observed in NLRP3, CASP7, and IRF1. Of these 51 patients, one patient had an NLRP3 mutation (2%), one patient had a CASP7 mutation (2%), and one patient had an IRF1 mutation (2%), taken together, gives a PRG mutation rate of (5.88%). Frame shift was detected in CASP7, missense mutation was detected in NLRP3 and IRF1, and the mutations in these two genes occurred mainly in their respective functional structural domains (Fig. 1A and B). The dominant copy number variation (CNV) type of AIM2, PARP1, and NLRP3 was gain of function, and that of TNFAIP3, TAB2, and TAB3 was loss of function (Fig. 1C). The positions of CNV alterations for PRGs were observed on human chromosome 23 (Fig. 1D). Fourteen PRGs were differentially expressed between the BTC and adjacent normal samples; among which, CASP1, CASP6, CASP7, CASP8, FADD, GSDMD, IRF1, MLKL, PARP1, RIPK1, RIPK3, TAB3, TNFAIP3, and TRADD were upregulated in the tumor samples (*p* < 0.05) (Fig. 1E). We further investigated the effect of 19 PRGs on the prognosis of patients with BTC based on the TCGA’s clinical information (Figure S1). The patients with low MLKL and RIPK3 expression had a better prognosis.

### Identification of molecular subtypes based on PRGs in patients with BTC

**Fig. 2:**
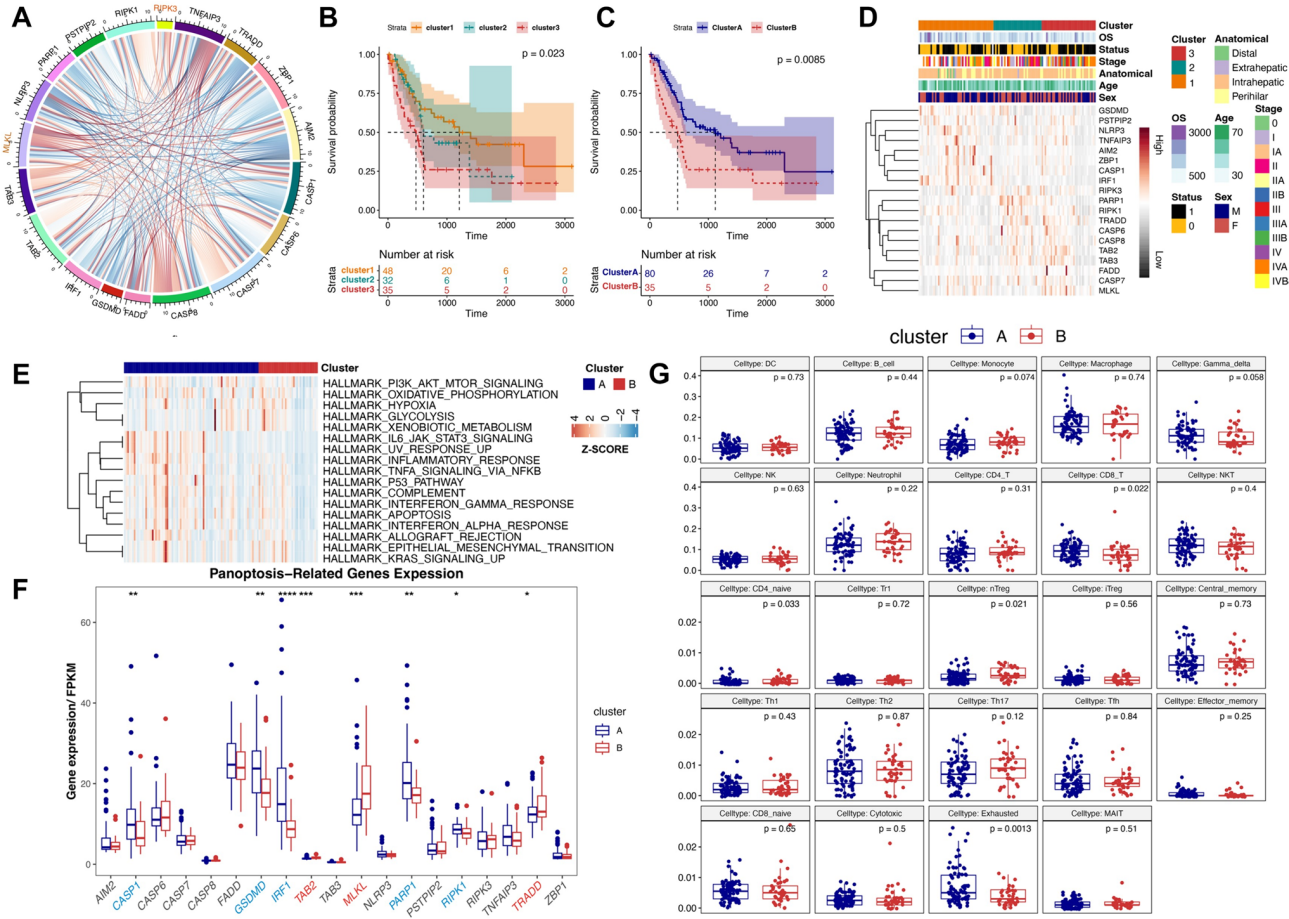
Identification of molecular subtypes based on PRGs in patients with BTC. **A** Pearson’s correlation circle plot of 19 PRGs. **B** Differential OS and risk curve between Clusters 1–3 (*k* = 3). **C** Differential OS and risk curve between Clusters A (Clusters 1–2) and B (Cluster 3). **D** Heatmap showing specific information of Clusters 1–3 such as OS, stage, age, sex, and expression of the 19 PRGs. **E** Differential signaling pathway enrichment in Clusters A and B based on HALLMARK. **F** Differential PRGs expression in Clusters A and B. **G** Difference in abundance among 24 immune cells in Clusters A and B. *p < 0.05; **p < 0.01; ***p < 0.001

We analyzed the correlation between the 19 PRGs according to their expression in the TCGA database by Pearson correlation coefficient (Fig. 2A) and selected GSE89749 with many cases to further analyze the relationship between PRGs and BTC. We performed consensus clustering analysis based on the expression of PRGs. Figures S2 and S3 show the clustering of samples at *k* values of 2–6, demonstrating the different modules produced at different *k* values (Figures S2 and S3). Significant difference in survival was observed among the three cluster samples divided by consensus clustering analysis at *k* = 3 (*p* = 0.023). We uniformly categorized Clusters 1 and 2 as Cluster A and Cluster3 as Cluster B in accordance with the survival curves. Further analysis revealed that the statistical difference (*p* = 0.0085) between the two groups of survival was highly pronounced (Fig. 2B and C). In addition, we showed the clustering by consensus clustering analysis for *k* = 2 and the results of survival and PCA analysis (Figure S4A and B). The results of PCA analysis on Clusters 1–3 or Cluster A and B are shown in Figure S4C and D. We further presented the specific information of Clusters 1–3 (*k* = 3) and Clusters 1–2 (*k* = 2) such as OS, stage, age, sex, and expression of the 19 PRGs using a heat map (Figs. 2D and S4E). Gene Set Variation Analysis (GSVA) demonstrated that some classical pathways such as the AKT signaling pathway were significantly upregulated in Cluster B. However the activation of the p53 pathway, a classical oncogenic pathway, was positively correlated with the gene expression levels in Cluster A dataset, which was also consistent with the trend of prognostic differences between the two groups (Fig. 2E). CASP1, GSDMD, IRF1, PARP1, and RIPK1 were highly expressed in Cluster A, and TAB2, MLKL, and TRADD were highly expressed in Cluster B (Fig. 2F). We further analyzed the difference between Clusters A and B based on the infiltration score in the Immune Cell Abundance Identifier (ImmuCellAI) and found less immune cells infiltrating the subgroup (Cluster B) with poor prognosis (Figure S4F). We further explored the difference of 24 immune cells abundances in Clusters A and B (Fig. 2G), where the 24 immune cells were composed of 18 T-cell subtypes and 6 other immune cells, namely, B cells, NK cells, monocyte cells, macrophages, neutrophils, and DC cells. We found that the cell type of CD4_naïve and nTreg were significantly enriched in Cluster B (*p* = 0.033 and 0.021). In Cluster A, the quantities of Exhausted cell type were higher than those in Cluster B (*p* = 0.0013).

### Differential expression genes analysis and signaling pathway enrichment for patients with BTC stratified by PRG clusters

**Fig. 3:**
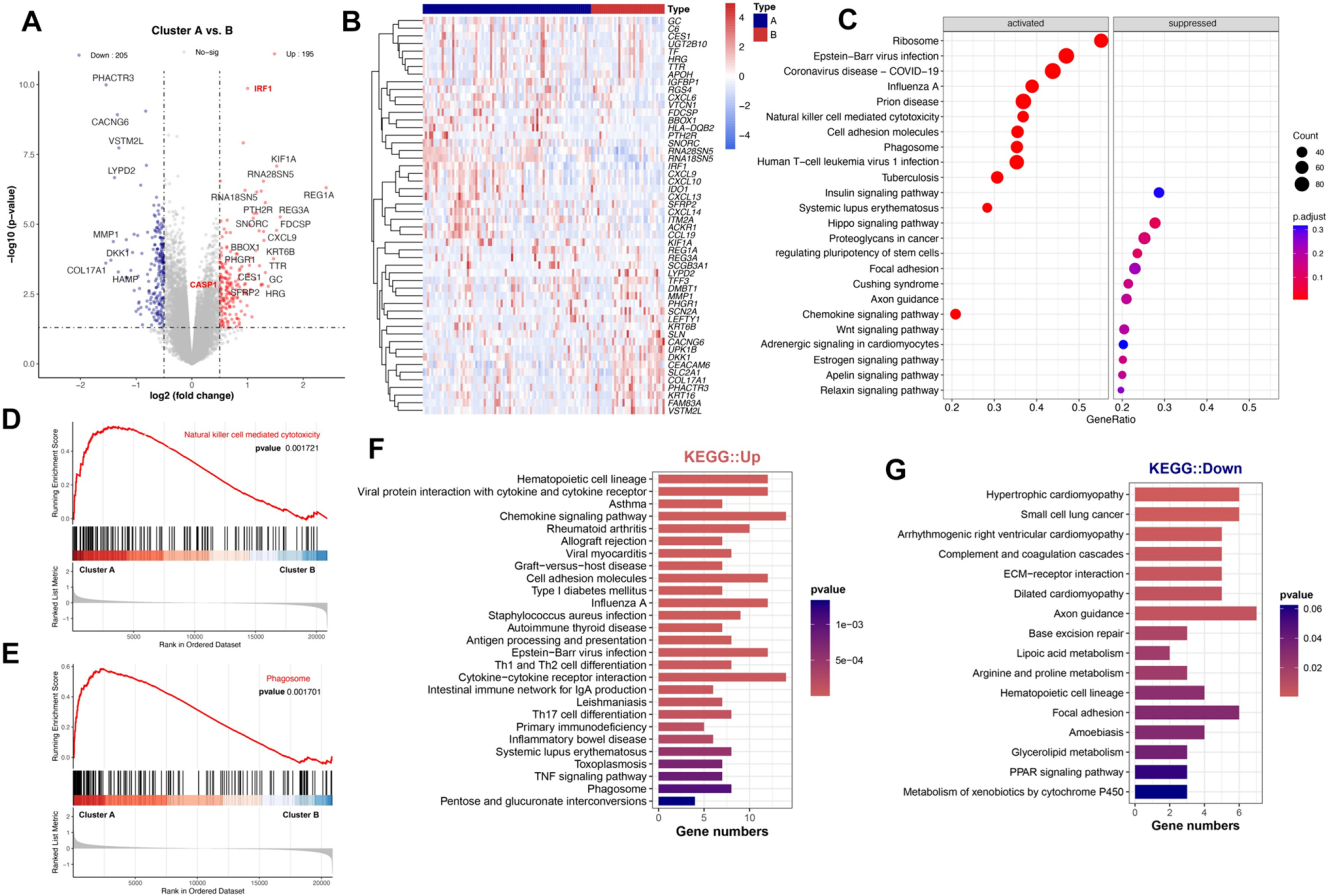
Differential expression genes analysis and signaling pathway enrichment for patients with BTC stratified by PRG clusters. **A** and **B** Differential analysis of PRGs in Clusters A and B by DESeq2. **C**–**E** GSEA functional enrichment analyses of Clusters A and B. **F** and **G** KEGG functional enrichment analyses of cluster A and B. *p < 0.05; **p < 0.01; ***p < 0.001

Differential analysis of PRGs for Clusters A and B revealed 400 differential expressed genes (DEGs) by DESeq2 (Fig. 3A and B ). Among them, 195 genes such as the key genes IRF1 and CASP1 in the PRGs mentioned earlier were upregulated in Cluster A, and 205 genes were downregulated in Cluster A. GSEA functional enrichment analyses (Figs. 3C) revealed the main signaling pathway difference in the PRGs of Clusters A and B. We observed that several infection-related pathways actively enriched in cluster A whereas several endocrine pathways enriched in cluster B. Thereout, we inferred that alterations in these bioprocesses may be the underlies influencing the PANoptosis condition of BTC patients. We found that the natural killer cell-mediated cytotoxicity pathway and the phagosome pathway were significantly activated in Cluster A (Figs. 3D and E). KEGG functional enrichment analyses revealed that the DEGs were enriched in the following main signaling pathways: Th17 cell differentiation, TNF signaling pathway, phagosome, ECM–receptor interaction, and focal adhesion. These pathways were strongly associated with malignant phenotypic transformation, invasive metastasis, and poor BTC prognosis [30–33].

### Construction of the 9-gene risk score prognostic model based on PRGs

**Fig. 4:**
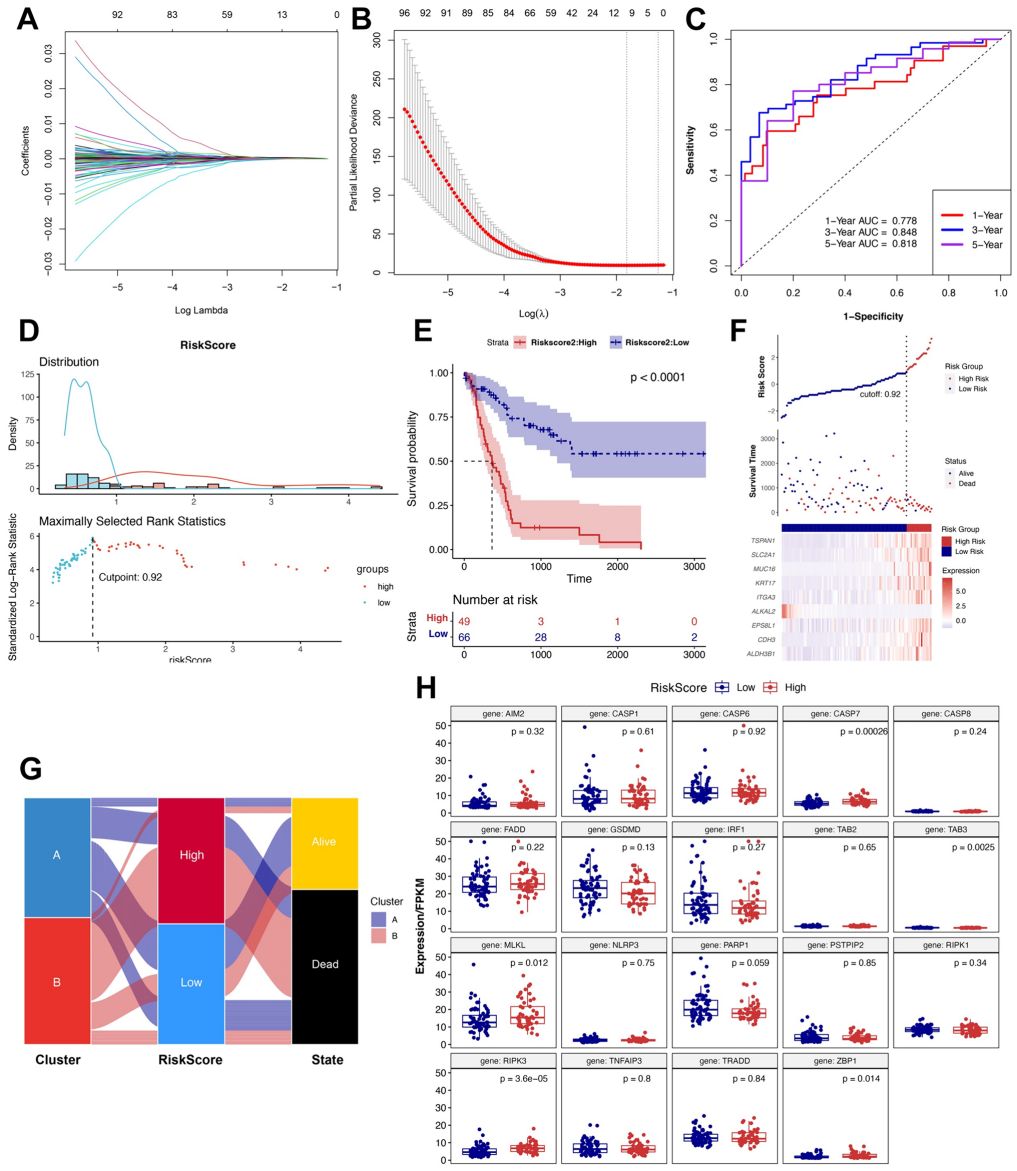
Construction of the 9-gene risk score prognostic model based on PRGs. **A** Lasso coefficient profiles of the differential genes in Clusters A and B. **B** Identification of the best Lambda value. **C** ROC curves used to compare the predictive performance for prob-min and prob-1se to predict the 1-/3-/5-year survival of patients with BTC. **D** Identification of the cutoff value (cutpoint = 0.92) of the risk score. **E** Patients with BTC were divided into high- and low-risk groups based on the cutoff value. Kaplan–Meier survival curves showing the difference in OS between high- and low-risk patients with BTC. **F**, **G** Survival status, survival times, and gene models in high- and low-risk groups. **H** Expression of 19 PRGs in high- and low-risk patients with BTC. *p < 0.05; **p < 0.01; ***p < 0.001

We calculated the coefficient values at different levels of penalty (Fig. 4A). First, we identified the optimal lambda (*λ*) value based on tenfold cross-validation. We selected two best-fit values (lambda. min and lambda.1se) by minimizing the mean-square error to construct the Lasso models and then chose two groups of genes (9-gene group of λmin and 4-gene group of λ1se, Fig. 4B). To enhance the model performance of prognostic prediction, we ultimately chose 9-gene risk predictive model to further explore. ROC curve analysis for the predictive models showed the AUCs were 0.818 (5 year), 0.848 (3 year), and 0.778 (1 year), suggesting that the models had a promising performance in predicting the probability of overall survival (Fig. 4C). The risk scores predicted by the coefficient of these nine candidate genes from the multivariate Cox regression analysis stratified the patients into low- (*n* = 66) and high-risk (*n* = 49) groups with a cut-off point of 0.92 (Fig. 4D). Kaplan–Meier survival curves showed the difference (*p* < 0.0001) in OS between the high- and low-risk patients with BTC (Fig. 4E). The expression of the nine genes in the high- and low-risk groups is shown in Fig. 4F. The relationship among the cluster, risk score, and survival status is visualized in Fig. 4G. Most of the patients with BTC in Cluster B had a high-risk score and a poor prognosis. We compared the differences in the expression levels of the 19 PRGs in the high- and low-risk groups and found that the expression levels of CASP7, TAB3, MLKL, RIPK3, and ZBP1 were statistically different between the two groups (Fig. 4H).

### Validation of the 9-gene risk score prognostic model and development of predictive nomogram for prognosis prediction

**Fig. 5:**
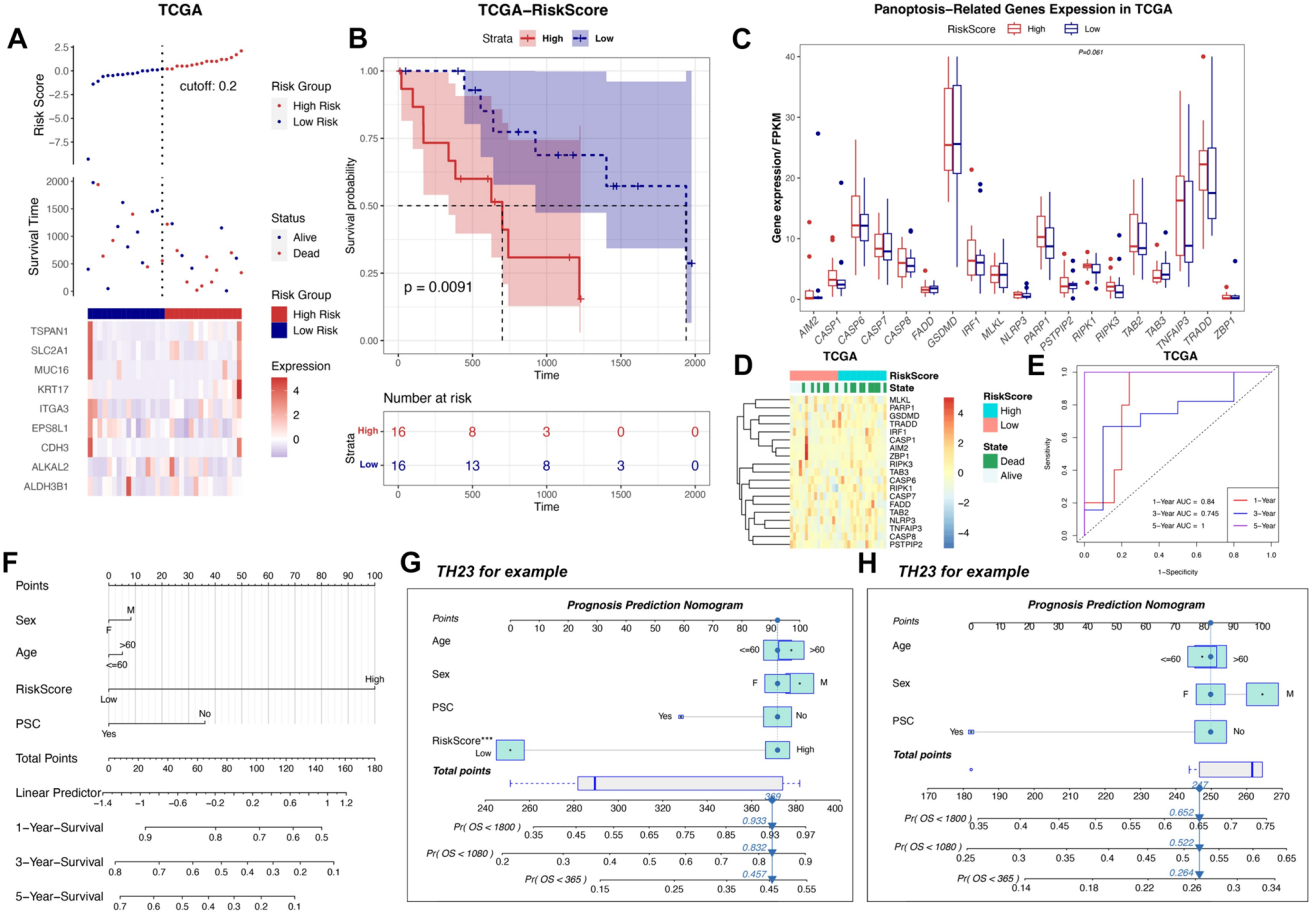
Validation of the 9-gene risk score prognostic model and development of predictive nomogram for prognosis prediction. **A** Identification of the cutoff value (cutpoint = 0.2) of the risk score in the external validation cohort and the heatmap of the 9-gene panel. **B** Differential OS and risk curve between the high- and low-risk groups in the external validation cohort. **C** PRG expression in the high- and low-risk groups in the TCGA validation cohort. **D** Heatmap of the risk score, survival state, and expression of 19 PRGs. **E** Validation of the 9-gene risk score predicting the AUC values for 1 year, 3 year, and 5 year OS of patients with BTC based on TCGA datasets. **F** Predictive nomogram was constructed by combining the risk scores with age, sex, and PSC. **G** and **H** Predictive results of TH23 for example with or without risk-score. *p < 0.05; **p < 0.01; ***p < 0.001

External validation was performed to clarify the generalizability of the 9-gene risk score prognostic model. The TCGA BTC cohort was divided into high- and low-risk groups according to the risk score (cutoff = 0.2, Fig. 5A). As shown in Fig. 5B, the high-risk group exhibited poor OS (*p* = 0.0091). In the TCGA validation cohort (Fig. 5C), the difference in RIPK1 expression between these two groups almost showed statistical significance (*p* = 0.061). This phenomenon may be related to the small sample size of the validation group. The heat map of the risk score, survival status, and expression of 19 PRGs is shown in Fig. 5D. The AUC values of the 9-gene risk score prognostic model for predicting the 1 year, 3 year, and 5 year OS of patients with BTC were promising (Fig. 5E). Independent prognostic analysis (Figure S5A–J), nomogram (Fig. 5F), and clinical applicability analysis (Fig. 5G and H) confirmed that the 9-gene risk score prognostic model could independently, accurately, and individually predict the survival of patients in the BTC cohort for a wide range of populations. We selected TH23 patients from the GSE89749 database for randomization validation and found a large difference in prediction accuracy after using or not using the risk score as a nomogram factor prediction (Fig. 5G and H).

### Immune landscape based on the 9-gene risk score and prediction of tumor response to immunotherapy and chemotherapy

**Fig. 6:**
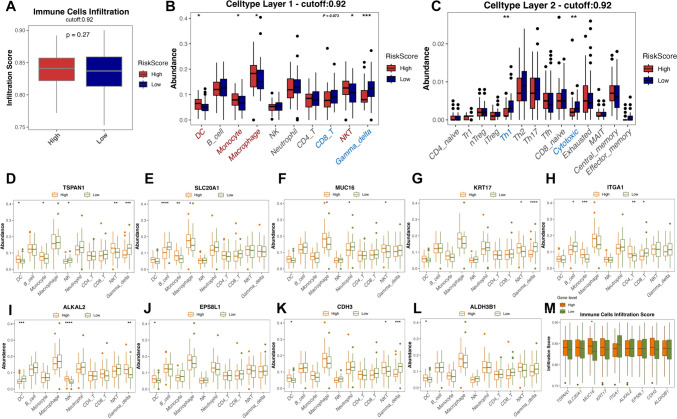
Immune landscape based on the 9-gene risk score and prediction of tumor response to immunotherapy and chemotherapy. **A** Immune cell infiltration levels between the two risk groups in the GSE89749 cohort. **B** and **C** Differential immune cell expression in the two risk groups. **D**–**L** Relationship between the expression of the nine genes and different immune cell subtypes. **M** Relationship between the expression of the nine genes and immune cell infiltration score. *p < 0.05; **p < 0.01; ***p < 0.001

We next assessed the association between the risk score and immune cell infiltration based on GSE89749. Analysis revealed no difference in infiltration score in ImmuCellAI between the high- and low-risk groups (Fig. 6A). We then explored the differential immune cell abundance of cell types in layers 1 and 2 for the high- and low-risk groups (Fig. 6B and C). The number of DC cells, monocytes, macrophages, and NKT cells was high in the high-risk groups. Meanwhile, CD8 T cells, gamma-delta cells, Th1 cells, and cytotoxic cells were abundant in the low-risk patients. The samples were further grouped based on the high or low expression of the nine genes, and the abundance of various immune cell infiltrations was analyzed at the subgroup level (Fig. 6D–L). We found that each risk gene was correlated with different immune cell subtypes. Furthermore, we correlated the expression level of each risk gene with the immune cell infiltration score and found that MUC16 was significantly correlated with the immune cell infiltration score (Fig. 6M), implying its critical role in the 9-gene risk score model. In addition, we investigated the relationship between chemotherapeutic agents and risk score models. We used the “pRRophetic” R package to predict the responses to 12 common chemotherapeutic agents from gene expression data (prediction of clinical outcomes based on Cancer Genome Project CGP Cell Line Data). Docetaxel and vinblastine were found to be significantly different in the two groups (Figure S5K and L). In the future treatment for BTC, low-risk patients may benefit from chemotherapy based on docetaxel or vinblastine and immunotherapy. Finally, we downloaded single-cell data from 5 cases of ICC tumor tissue provided in the GSE138709 dataset. After screening and filtering, 4964 cells were obtained for subsequent analysis (Figure S6A-D). 5 samples were merged by the CCA algorithm provided by Seurat for inter-sample merging and batch effects removal, and the cells were downscaled for visualization using UMAP. Cells were randomly classified into 12 clusters from 0 to 11, and the left panel shows the coloring of cells according to their clusters. We then annotated the cells (Figure S6E). Demonstration of cellular annotation Marker gene expression in each cluster of cells after annotation (based on GeneSet provided in the original GSE138709) (Figure S6F). UMAP projection of cells after annotation, with malignant cells predominantly in the samples, and some immune cells, mesenchymal stromal cells and a small number of bile duct epithelial cells were also detected (Figure S6G). We then examined the expression of nine genes in each cell, of which ALKAL2 expression was not detected in this single-cell data, resulting in a total of eight genes (Figure S6H). Four of them had significant expression in immune cells, SLC20A1 with KRT17 was more expressed in malignant tumor cells (Figure S6I and J) and ITGA1 was more expressed in mesenchymal cells (Figure S6K and L).

## Discussion

BTC is a heterogeneous group of tumors that are classified as intrahepatic, perihilar, distal, or gallbladder depending on their anatomic location within the biliary tract [34]. Surgical resection is the best treatment for patients with BTC. Owing to the insidious onset of the disease and the lack of specific tumor markers, the majority of patients with BTC are already at an advanced stage once detected and suitable treatments are lacking [35]. PANoptosis is an inflammatory PCD regulated by the panapoptotic vesicle complex and has the key features of cellular pyroptosis, apoptosis, and necroptosis, making it a hotspot and focus of PCD research in the future [36]. PANoptosis is closely related to infectious diseases, tumors, and other human diseases. Although its specific regulatory mechanism has not been elucidated, PANoptosis has already shown good research prospect as a potential target for intervention [37]. A comprehensive understanding of the role of PANoptosis in human diseases will help elucidate its regulatory mechanism for exploring a new therapeutic strategy and achieving breakthroughs in the treatment of related diseases [11, 36]. To date, no studies have focused on PANoptosis in biliary malignancies. Therefore, if some markers for predicting the prognosis and adjuvant treatment efficacy of BTC are screened by PANoptosis panel, then it may improve the level of treatment for these patients [23, 38].

The majority of the 19 PRGs discussed in our study were related to the occurrence and development of BTC. High MLKL and RIPK3 expression predicts poor prognosis. RIPK1, RIPK3, and MLKL-p expression have relevance to the OS of patients with HCC [39]. Lomphithak T [40] explored the relationship of MLKL with the clinicopathological characteristics, survival data, and prognosis of patients with BTC and found it to be an adverse prognostic factor and was inversely correlated with a clinically favorable immune cell signature (high CD8^+^/high FOXp3^+^/low CD163^+^). These data were consistent with our results about the differential expression of PRGs between adjacent normal samples and BTC samples. Given the relevance of BTC classification to PRG expression, our research showed that the prognosis of PRG cluster B showed more negative results than that of PRG cluster A with its enrichment of immune-related pathways and immune cell infiltration. KEGG functional enrichment analyses revealed that the DEGs were enriched in the following main signaling pathways: Th17 cell differentiation, TNF signaling pathway, phagosome, ECM–receptor interaction, and focal adhesion. Kinoshita M [30] reported that IL-6 and TGF-β1 secreted by BTC cells promoted the derivation of Tregs and Th17 cells, and Th17 cells accumulated at the tumor margin to accelerate the inflammatory response and promote tumor cell invasion. With regard to the ECM–receptor interaction and focal adhesion, Song [41] found that focal adhesion kinase promoted cholangiocarcinoma development and progression via YAP activation. Hong [42] demonstrated that CCR7 mediated the TNF-α-induced lymphatic metastasis of gallbladder cancer through the “ERK1/2-AP-1” and “JNK-AP-1” pathways.

The majority of current studies on PANoptosis in tumors focused on solid malignancies such as hepatocellular carcinoma [12–14], pancreatic carcinoma [15], lung carcinoma [16–18], and renal carcinoma [19, 20]. However, only a few reports on BTC are available. Zhang [15] reported that PANoptosis-related molecular subtype and prognostic model could predict the prognosis of patients with pancreatic cancer. Shi [12] constructed a PANoptosis-related gene model and characterized the tumor microenvironment infiltration in hepatocellular carcinoma. Wang [19] explored a novel-defined PANoptosis-related miRNA signature for predicting the prognosis and immune characteristics in clear cell renal cell carcinoma. In our study, we constructed a 9-gene risk score prognostic model based on PRGs for predicting the prognosis of patients with BTC. ROC curve analysis for the predictive models showed the AUCs were 0.818 (5 year), 0.848 (3 year), and 0.778 (1 year), indicating that the models had a promising performance in predicting the probability of overall survival. Zhang [43] reported that the AUCs of 1-, 3-, and 5-year survival periods were 0.632, 0.665, and 0.707, respectively. The PANRS based on the PANoptosis panel constructed by Zhu and his colleagues predicted the AUC values for 1-, 3-, and 5-year OS of patients with hepatocellular carcinoma to be 0.793, 0.720, and 0.709, respectively [44]. These results verified the credibility of our data and indicated that the PANoptosis-related gene set can be used for predicting the prognosis of BTC and enriches the BTC-related biomarkers.

Immune checkpoint molecules prevent the abnormal activation of immune responses and maintain homeostasis in the body [45, 46]. However, tumor cells take advantage of this property of immune checkpoint molecules to evade the immune response [47]. Therefore, immune checkpoint inhibitors (ICIs) for mitigating immune evasion are the fourth most common form of cancer treatment after surgery, chemotherapy, and radiotherapy [48, 49]. The number of DC cells, monocytes, macrophages, and NKT cells was high in the high-risk groups. Our research suggested that the high-risk group with increased expression of immune checkpoint genes may have a favorable response to ICI therapy. The number of CD8 T cells, gamma-delta cells, Th1 cells, and cytotoxic cells was high in the low-risk patients. The expression levels of these cells determined the efficacy of immunotherapy [49, 50]. The low abundance of CD8 T cells, gamma-delta cells, Th1 cells, and cytotoxic cells suggested an increased probability of tumor immune evasion [47–49]. We found that each risk gene was correlated with different immune cell subtypes. MUC16 may play a critical role in the 9-gene risk score model. Heczko et al [51] demonstrated that patients with colon cancer and somatic variants in VIPR2 had significantly shorter OS, and those with alterations in MUC16 had longer OS. Zhang reported that the genes MET, MUC16, and KRT7 in the model had high expression levels in pancreatic tumor tissues as validated by RT-qPCR. Furthermore, single-cell analysis revealed that MET, MUC16, and KRT7 were mainly expressed in the microenvironment of pancreatic cancer cells [52]. All of these results suggested that MUC16 shows potential as a marker for the malignant phenotypic transformation of malignant tumors. In the past, the therapeutic approach to systemic treatment of BTC was restricted to the use of chemotherapy. For more than a decade, the combination of cisplatin and gemcitabine has been the standard of care in the first-line systemic treatment of BTC. However, the recent development of immunotherapy and targeted therapy has rapidly changed the landscape of BTC treatment and revolutionised the therapeutic to this aggressive malignancy in the advanced stage [53]. In the results of Supplementary Fig. 5K and L, it is true that docetaxel or vinblastine may be of higher benefit in the low-risk group, but because of the greater overall immune infiltration in the high-risk group, giving the appropriate immunotherapy is a promising therapeutic prospect, and the specifics will need to be confirmed with more patient samples as well as single-cell data. Therefore, new targets for BTC therapy screened by inhibition of PANoptosis-related signature genes and combined with chemotherapy have the potential to improve the prognosis of BTC patients. We identified a number of patient groups potentially suitable for chemotherapy based on PRGs, but this finding requires in vivo and in vitro experiments for validation in the future.

Some limitations existed in our research. First, the data were obtained from public databases (such as TCGA or GEO datasets). In the future, we will recruit random prospective samples. Second, the inclusion of clinicopathological indicators was not comprehensive enough; actual clinical samples are needed to evaluate our results. In summary, analysis using the combination of bioinformatics and molecular biology suggested PANoptosis to be associated with BTC-related prognosis and immunity. Nine genes based on PRGs were identified as potential biomarkers for diagnosis and treatment. This research provided us with a profound understanding of PANoptosis in BTC and promising strategies for the clinical therapy of BTC.

## Conclusions

PRGs can accurately predict the prognosis and immunotherapy response of patients with BTC and consequently guide individualized treatment. This research provided us with a profound understanding of PANoptosis in BTC and promising strategies for the clinical therapy of BTC.

## Supplementary Information

Below is the link to the electronic supplementary material.

Supplementary file1 (DOCX 4536 KB)

## Data Availability

The code of our proposed method is published at https://github.com/XingtianXing/PANoptosis

## Abbreviations

BTC: Biliary tract cancer
HCC: Hepatocellular carcinoma
CCA: Cholangiocarcinoma
PCD: Programmed cell death
PRG: PANoptosis-related gene
GEO: Gene expression omnibus
TCGA: The cancer genome atlas
DEG: Differentially expressed gene
KEGG: Kyoto encyclopedia of genes and genomes
GO: Gene ontology
LASSO: Least absolute shrinkage and selection operator
KM: Kaplan–meier
AUC: Area under the curve
ROC: Receiver operating characteristic
PSC: Primary sclerosing cholangitis
TME: Tumor microenvironment
CNV: Copy number variation
PCA: Principal component analysis
OS: Overall survival
GSVA: Gene set variation analysis
ImmuCellAI: Immune cell abundance identifier
ICI: Immune checkpoint inhibitor
