# Supplementary material for: Identification of PANoptosis-relevant subgroups and predicting signature to evaluate the prognosis and immune landscape of patients with biliary tract cancer
Source: Hepatol Int. 2024 Aug 10;18(6):1792–803. doi: 10.1007/s12072-024-10718-x (PMC11632078; doi:10.1007/s12072-024-10718-x)

**Supplementary Figure 1.**

19 PRGs on the prognosis of patients with BTC based on the TCGA’s clinical information


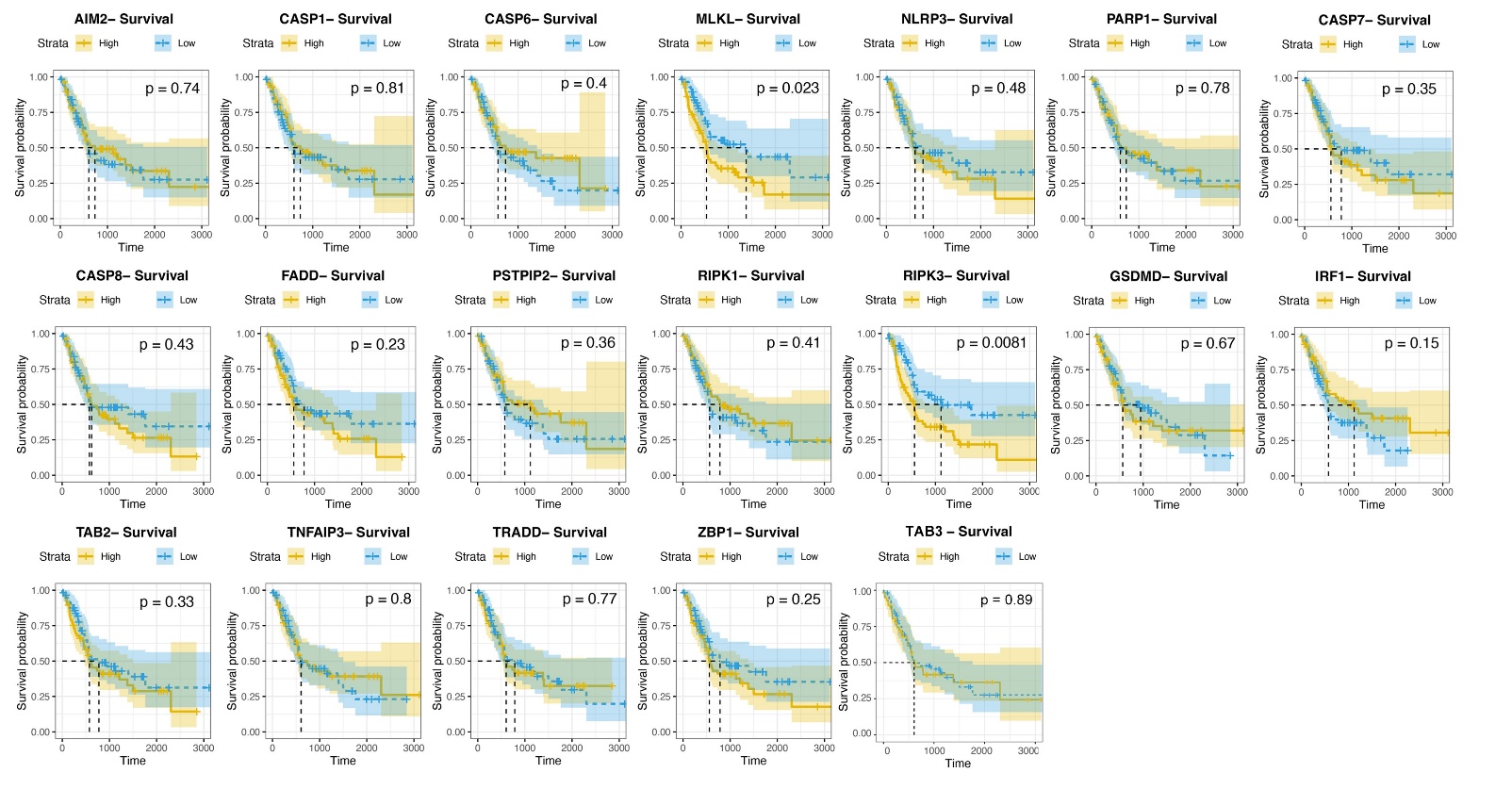


**Supplementary Figure 2 and Figure 3.**

Clustering of samples at k values of 2–6, demonstrating the different modules produced at different k values

**
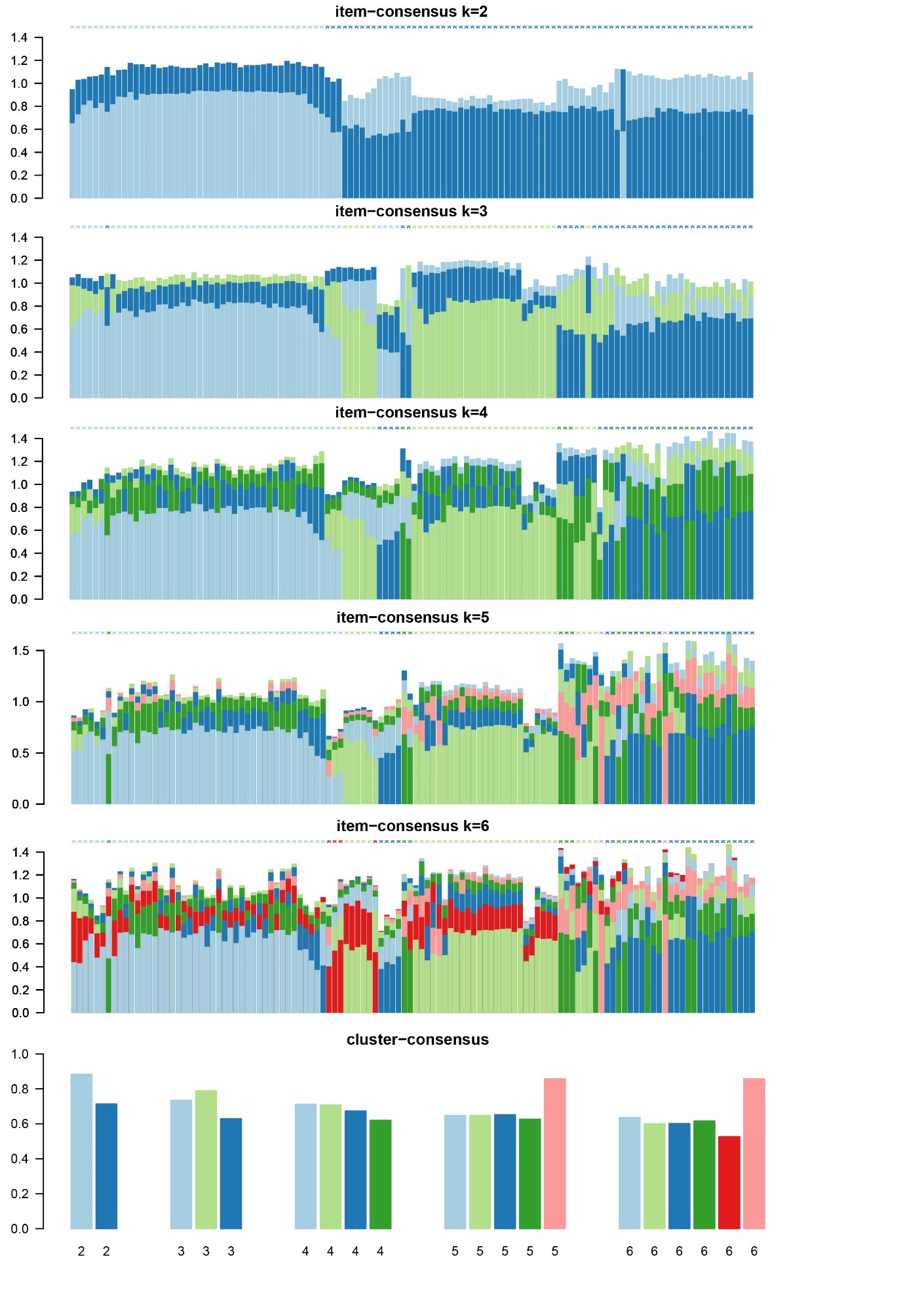
**

**
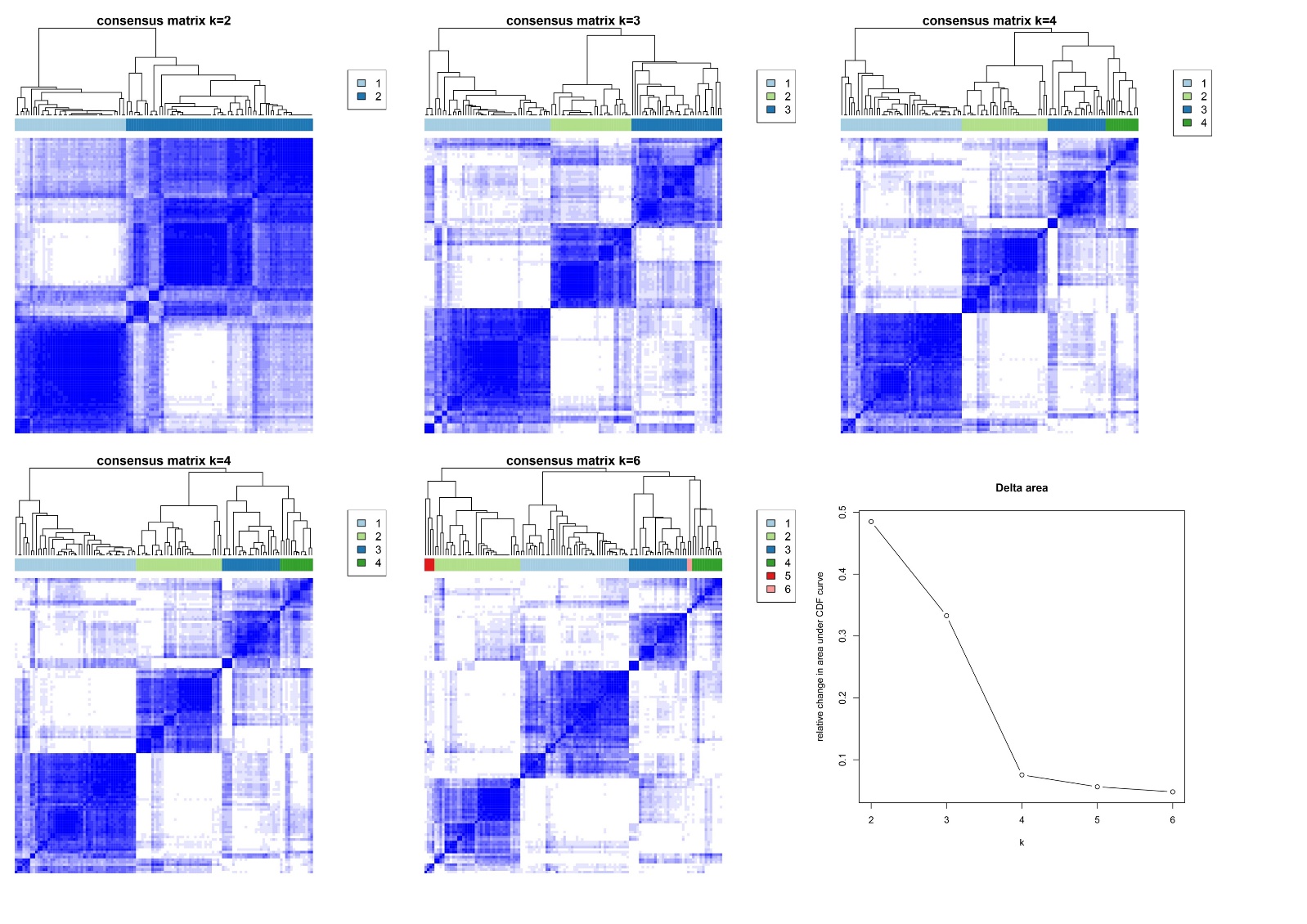
**

**Supplementary Figure 4.**

(A, B). Clustering by consensus clustering analysis for k=2 and the results of PCA analysis. (C, D). The results of PCA analysis on Clusters 1-3 or Cluster A and B. (E). Clusters 1-2 (k=2) such as OS, stage, age, sex, and expression of the 19 PRGs using a heat map. (E). The difference between Clusters A and B based on the infiltration score in Immune Cell Abundance Identifier (ImmuCellAI). ^ns^P > 0.05, **P* < 0.05, ***P* < 0.01, ****P* < 0.001.


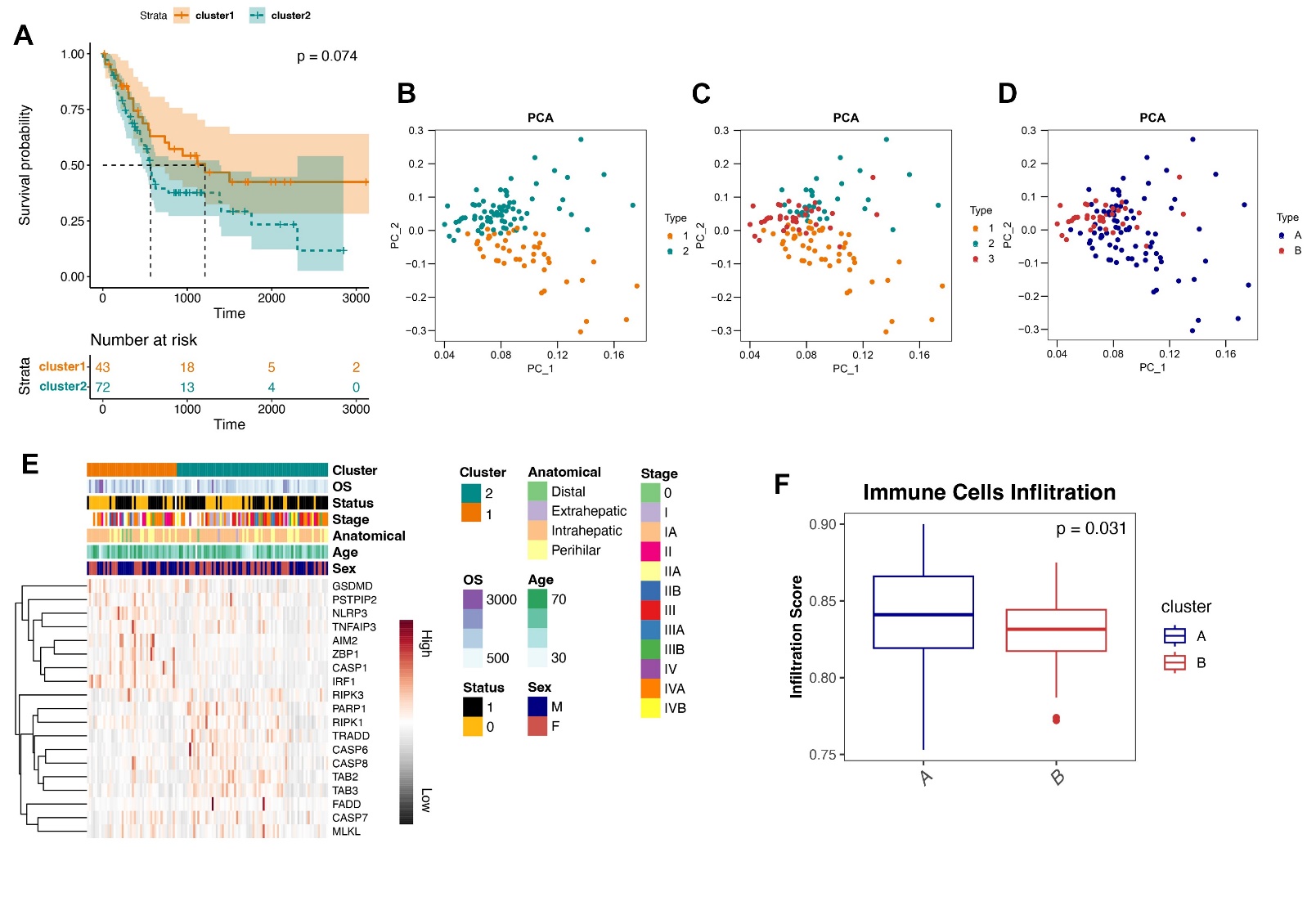


**Supplementary Figure 5.**

(A-J). Independent prognostic analysis of 9-gene risk score prognostic model. (K, L). Predicting the responses to 12 common chemotherapeutic agents from gene expression data. **P* < 0.05, ***P* < 0.01, ****P* < 0.001.


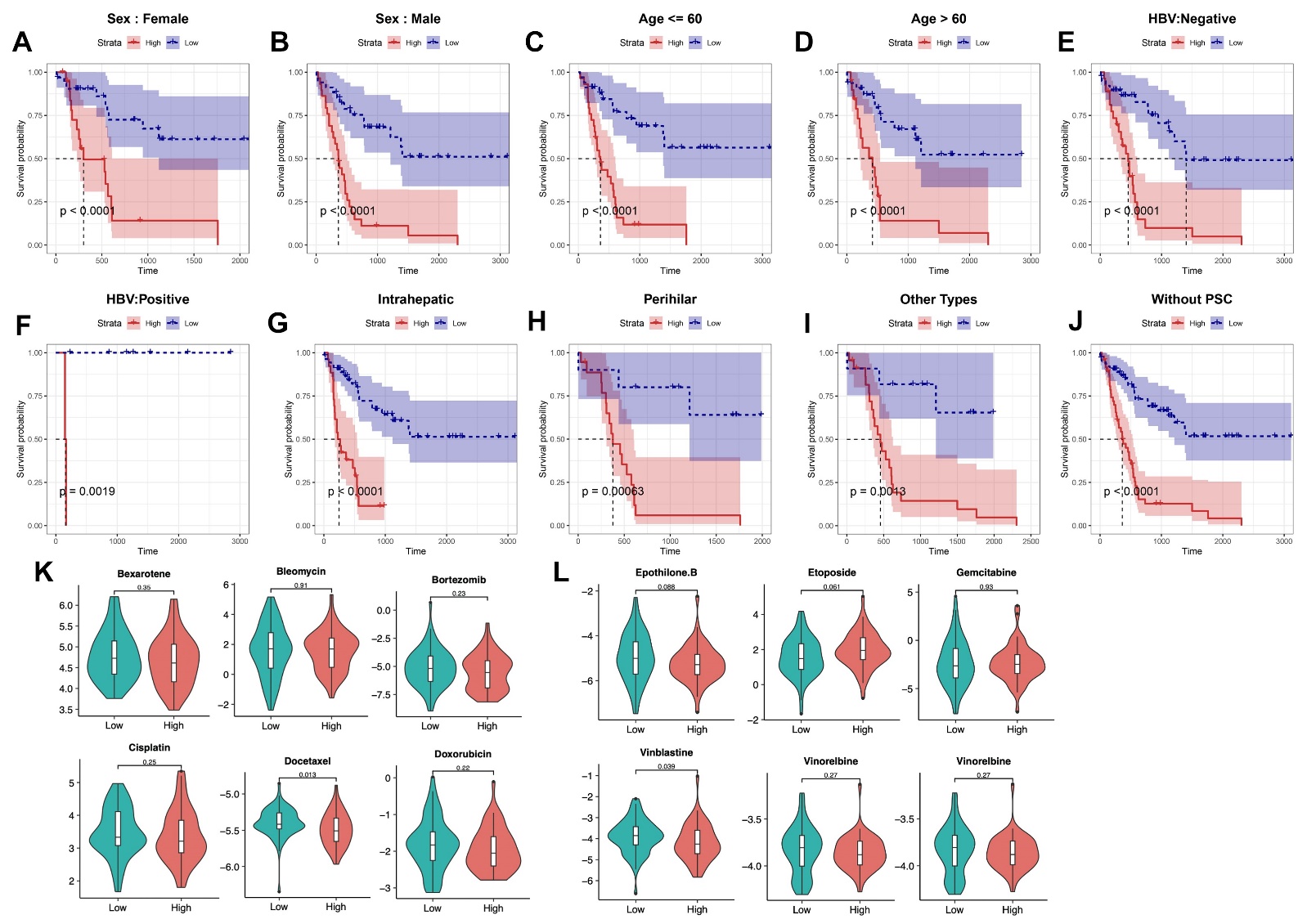


**Supplementary Figure 6.**

The validation of the relationship between immune cell infiltration and nine-gene expression by using single-cell RNA sequencing public data

(A-D). After screening and filtering, 4964 cells were obtained for subsequent analysis. (E). Cells were randomly classified into 12 clusters from 0 to 11. (F). Violin plot: demonstration of cellular annotation Marker gene expression in each cluster of cells after annotation. (G). UMAP plot: UMAP projection of cells after annotation. (H). The expression of nine genes in each cell, of which ALKAL2 expression was not detected. (I-L). The significant expression in immune cells.


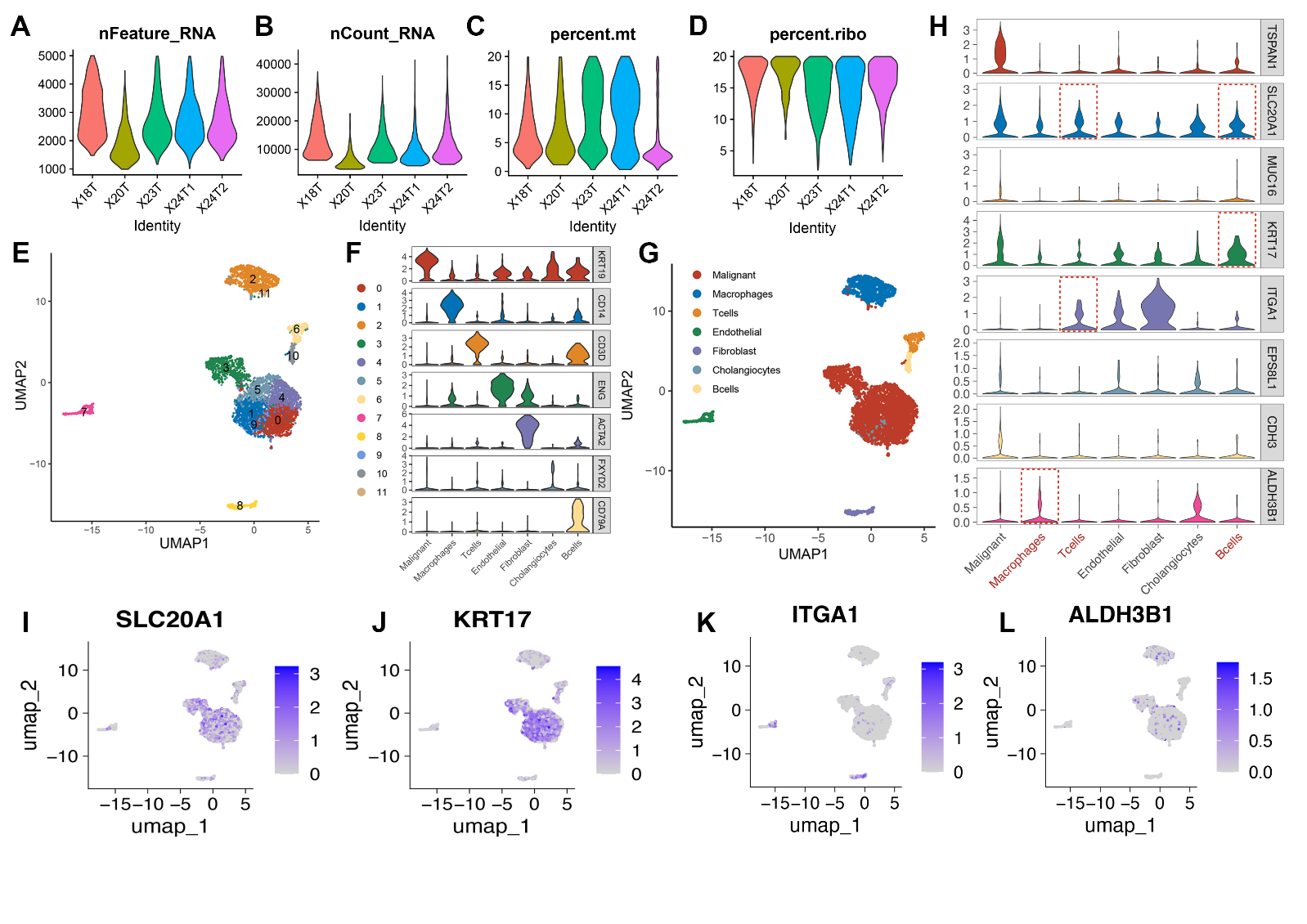


**Supplementary Figure 7.**

The flow chart of this study.


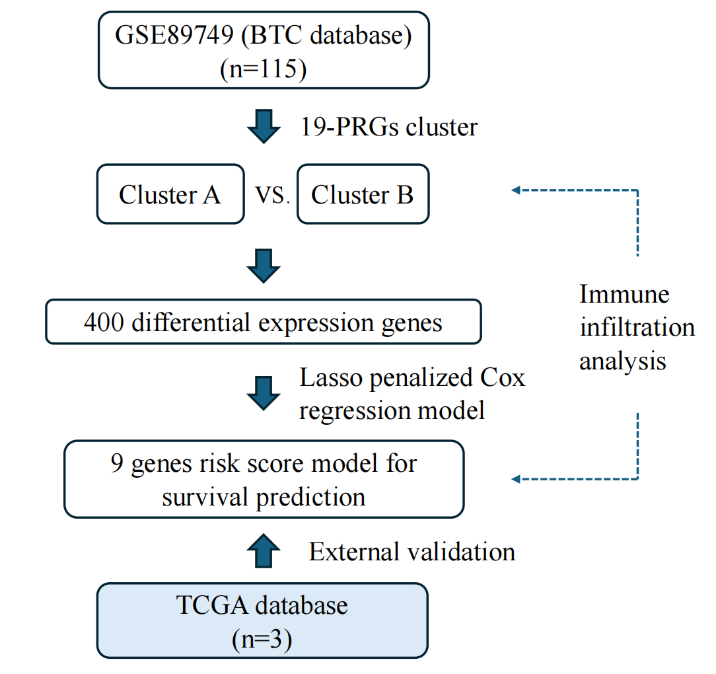

Supplement: Supplementary file 1 — Supplementary file1 (DOCX 4536 KB) [file 12072_2024_10718_MOESM1_ESM.docx]
